# Ethical Considerations of Climate Justice and International Air Travel in Short-Term Electives in Global Health

**DOI:** 10.4269/ajtmh.22-0508

**Published:** 2023-08-07

**Authors:** Adriana Dhawan, Zoe Rammelkamp, Johnstone Kayandabila, Vishnu Laalitha Surapaneni

**Affiliations:** ^1^ Division of Hospital Medicine, University of Minnesota, Minneapolis, Minnesota;; ^2^ Arusha Lutheran Medical Centre, Arusha, Tanzania;; ^3^ Eastern and Southern Africa Management Institute, Arusha, Tanzania;; ^4^ London School of Tropical Medicine & Hygiene, London, United Kingdom

## Abstract

In July 2022, the American Society of Tropical Medicine and Hygiene Green Task Force advocated to acknowledge the health impacts of climate change, particularly on those in low- and middle-income countries, and called on global health organizations to act. Simultaneously, academic medical centers are resuming Short-Term Electives in Global Health (STEGH) as travel restrictions imposed during the COVID-19 pandemic ease in most countries. International flights by trainees from academic medical centers in high-income countries (HIC) on these electives encapsulate the climate injustice of who generates carbon emissions and who bears the impacts of climate change. Using “decolonization” and “decarbonization” as guiding principles, we suggest several strategies that global medical education programs in HIC could implement. First, restructure rotations to halt STEGH with minimal benefit to host institutions, optimize trainee activities while abroad, and lengthen rotation duration. Second, programs can calculate the carbon impact of their STEGH and implement concrete measures to cut emissions. Finally, we urge academic medical centers to promote climate-resilient healthcare infrastructure in host countries and advocate for climate solutions on the global stage.

## INTRODUCTION

Short-Term Electives in Global Health (STEGH) are programs that typically facilitate volunteers and trainees from high-income countries (HIC) to travel to low- and middle-income countries to participate in a variety of programs, usually for a period of weeks.^1^ At academic medical centers, STEGH may be available for medical students, postgraduate trainees, or faculty in areas of clinical care, research, or public health. The COVID-19 pandemic halted STEGH, with many programs pivoting to maintain international collaborations virtually.^2^ As travel restrictions imposed during the COVID-19 pandemic ease in most countries, academic medical centers in high-income countries that facilitate STEGH are navigating how to reinstate rotations.

In general, the purpose of STEGH is primarily a practical educational experience for trainees, exposing them to health systems, medical conditions, and cultural contexts that differ from their home institution. The literature shows tangible benefits to trainees from HICs who engage in STEGH, ranging from physical exam and intercultural communication skills to cost-conscientiousness and resource allocation.^3^^–^^6^ There is value in collaborating with individuals with different medicocultural practices and engaging in dialogue that fosters a culture of inquiry. Importantly, STEGH can be career changing for participants, demonstrating associations with employment choices in public health and underserved primary care, and engagement in equity work.^4^^,^^7^^,^^8^

However, there are ethical concerns around multiple aspects of STEGH, including “medical voluntourism” and the need to decolonize medical global health education.^9^^–^^12^^,^^16^ As such, many educational programs are working to improve bilateral exchange and are revisiting long-standing practices in STEGH.^1^^,^^13^^–^^15^ Critical program review and pre-departure orientation education acknowledging problematic histories of colonialism and White saviorism are being implemented across U.S.-based academic medical centers.^10^^,^^16^^,^^17^ However, one consideration that has yet to feature prominently in the discussion about the harms and benefits of STEGH is climate change.

Climate change is the greatest threat to global public health in the 21st century, with stark inherent inequities.^18^ HICs have historically contributed the majority of emissions, leading to global warming. The United States, the single largest contributing country, emitted 25% of global cumulative CO_2_ emissions from 1751 to 2017, whereas Africa as an entire continent contributed 3%.^19^ Conversely, health impacts of climate change including sequelae of extreme heat, poor air quality, drought, flooding, and other extreme weather events are borne disproportionately by those who have contributed least to the problem – those in low-middle income countries, low-income communities, and Indigenous peoples.^20^^–^^22^ These same communities also suffer more from extractive industry processes and environmental pollution.^23^ To prevent worsening health impacts, it is clear that we need urgent action to reduce emissions.^24^

In response to the need for robust climate action, the American Society of Tropical Medicine and Hygiene (ASTMH) Green Task Force advocated that the global health community acknowledge the health impacts of climate change as part of its scope and engage in practices to address it.^25^ In this perspective piece, we focus on the role of international flights for STEGH from academic medical centers, which encapsulate the climate injustice of who generates emissions and who bears the impacts. Scientific and academic communities are already critically appraising whether international flights are necessary to facilitate productive conferences.^26^^,^^27^ Similarly, the Association of American Medical Colleges recommended medical residency interviews be conducted virtually in 2022–2023 after recognizing the carbon footprint generated by interview travel and other equity considerations.^28^^,^^29^ Aviation, a hard-to-decarbonize sector, is estimated to have contributed to 4% of global warming prepandemic through its CO_2_ (∼2.4%) and non-CO_2_ emissions.^30^^,^^31^ Although 4% may seem negligible, only a small fraction of the world’s population is responsible for it, highlighting flying as a privileged activity.^32^ Gössling and Humpe^33^ estimated that 1% of the world population emitted 50% of CO_2_ from commercial aviation. Within higher education institutions, air travel can contribute 30% or more of an institution’s carbon footprint.^34^^,^^35^^,^^36^

We argue that an ethical conflict of duty exists for academic medical centers that encourage, facilitate, and finance trainee travel on STEGH. Using “decolonization” and “decarbonization” as guiding principles (see Box 1), we reflect on the carbon impact of STEGH and how global health educators at academic medical centers can promote innovative strategies that embrace climate justice. Here we propose solutions to restructure rotations to align with these principles, acknowledge ecological impact, and engage in advocacy for public policy on climate.

Box 1 **Definitions**

Decolonization: In the context of global health education, decolonization “reaches beyond removal of colonial power and dismantling of colonial structures to include decolonization of the mind that made the colonizer feel superior and the colonized inferior by enforcing structural drivers of discrimination and barriers to self-determination.”^16^
Decarbonization: The process by which countries, individuals, or other entities aim to achieve zero fossil carbon existence. Typically refers to a reduction of carbon emissions at the source (Intergovernmental Panel on Climate Change).^37^
CO_2_ equivalent (CO_2_-eq) emission: The amount of carbon dioxide (CO_2_) emission that would cause the same integrated radiative forcing or temperature change, over a given time, as an emitted amount of a greenhouse gas (Intergovernmental Panel on Climate Change).^37^

## RESTRUCTURING ROTATIONS

### Review STEGH offerings.

The demand for STEGH from trainees has grown significantly over the past decades, with more than half of U.S.-based academic medical centers now offering international electives as a component of medical training.^4^^–^^6^^,^^38^ This appears driven by both globalization and increased interest from trainees in experiential learning opportunities, with programs adapting to draw applicants.^5^^,^^38^ There is significant variation in the structure of current STEGH. Academic medical centers should critically review their existing programs for high-quality characteristics such as bilateral, longitudinal relationships with host sites, robust pre-departure orientation, and on-site faculty support for trainees.^9^^,^^10^^,^^13^^,^^14^^,^^16^^,^^17^ Institutions whose STEGH offerings are sporadic, unsupported, and of a unilateral nature should consider the limited benefits to host partners and potential harms of continued rotations, including climate impact. Emulating the University of Pennsylvania, they should narrow STEGH offerings by halting international travel on rotations of limited benefit and reallocate support to quality programs, pivot to remote virtual collaboration, or reinvest in local underserved communities.^9^

### Activities abroad.

As noted earlier, academic medical centers should emphasize that STEGH relationships should not be extractive in medical training or climate impact. It is important to acknowledge that STEGH participants generally do not fill critical human resource gaps at host institutions and, when poorly executed, reflect “voluntourism,” impeding opportunities for local practitioners, undermining local health infrastructure, and potentially harming patients.^1^^,^^11^^,^^12^^,^^39^ If traveling, trainees should take part in research or clinical activities that require in-person presence as a component of longitudinal programs showing demonstrable benefit to host institutions and professional communities. Passive learning via observation and lecture-based programs should be reconsidered given developments in virtual capabilities. In collaboration with host partners, academic medical centers can outline participant expectations that optimize clinical engagement and define appropriate scope of practice for participants’ level of training prior to commencing STEGH placements.

### Rotation duration.

Currently, there is significant variation in the duration of STEGH during medical residency. Literature to support an optimal duration is sparse, with most studies on this topic found in pediatrics. For example, a 2016 study summarized that U.S. pediatric residency program electives ranged from 3 to 8 weeks in duration and could not identify the reasons for the recommended duration of 4 weeks.^40^ The American Board of Pediatrics publication *Global Health in Pediatric Education: An Implementation Guide for Program Directors* cited their expert opinion of 4 to 6 weeks in 2018, with a 4-week minimum.^7^^,^^41^ There appears to be no association with destination (e.g., distance traveled from sponsoring institution) and length of rotation. Current U.S. accreditation requirements largely ignore time spent outside the sponsoring institution toward minimum requirements for graduation, thus limiting rotation duration for most trainees. Others have advocated maximizing the duration of international electives at the individual rotation and accreditation levels.^4^^,^^40^ We strongly support these measures and would argue that contiguous time abroad not only facilitates a better clinical learning experience but minimizes emissions from multiple flights.^40^ Institutions sponsoring STEGH in distant host countries should consider greenhouse gas emissions and host partner needs when determining the frequency and minimum duration of the elective, and provide concrete support (e.g., administrative, financial) to host institutions to accommodate trainees for longer rotation durations.

## ACKNOWLEDGING ECOLOGICAL IMPACT

### Expanding carbon literacy.

Several medical schools across the United States are already integrating climate change into the curriculum.^42^^,^^43^ Global health programs, specifically, pre-departure programs, should highlight the geographically specific climate-health impacts being faced by the host country in addition to locally relevant history and decolonization practices. Education could include the carbon footprint of global health activities such as STEGH and ensuring learners are given tools to engage in sustainable behaviors. At the authors’ institution, for example, trainees participated in an interdisciplinary mock global climate negotiation exercise called “En-ROADS Climate Solutions Simulation” with a climate-policy expert and an economist. This training not only helped participants understand the disproportionate impacts of climate change and the need for urgent action but also highlighted science-based climate targets (as outlined by the IPCC’s 2018 Special Report on Global Warming of 1.5°C) and the impact of specific policies to reach them.^44^

### Internal analysis of carbon footprint.

Academic medical centers that facilitate any health-related or other international electives can conduct an internal analysis of their impact using available online tools. For example, a website is available that incorporates multiple calculators and methods to calculate flight emissions (https://travel-footprint-calculator.irap.omp.eu/), including radiative forces indices to account for non-CO_2_ effects.^45^ Using this tool, we estimate that between 2014 and March 2020, our global health program facilitated at least 126 metric tons of CO_2_-eq emissions through reimbursed trainee travel to our most frequented site in Tanzania alone.^45^ A majority of the 33 rotations were only 4 weeks long. Using the US Environmental Protection Agency and ClimateWatch databases, on average, a single trainee roundtrip flight contributed more than 1.5 times an average Tanzanian citizen’s yearly CO_2_-eq emissions.^46^^,^^47^ Travel to Tanzania was only approximately one-third of our global health program activities during this time, and institution-wide analysis including all sites and other exchange programs would reveal further eye-watering comparisons. Such analyses can not only serve as opportunities to reflect on climate injustice but also help generate short- and long-term emissions reduction targets and implementation plans to reduce an institution’s carbon footprint.

### Offsetting considerations.

Many commercial airlines offer optional carbon offsets for purchase through programs that plant trees, conserve forests, or fund renewable energy projects, thus theoretically negating the climate impacts of flights. Some universities have implemented offsetting schemes to variable effect.^35^^,^^36^ However, the authors recommend against academic medical centers purchasing offsets as a singular climate solution that allows programs to continue “business-as-usual” and ultimately fail in reducing greenhouse gas emissions.^48^^–^^50^ For example, in 2016, the European Union Commission concluded that 85% of the offsetting projects analyzed had a “low likelihood” of actual emissions reductions.^50^ The offsetting industry is complex, and concerns include lack of transparency and undervaluation at current price points.^51^^,^^52^ Case studies have also described how offset market-driven reforestation projects have displaced Indigenous peoples from their land, leading to the term “carbon colonialism.”^53^^,^^54^ Many frontline communities (i.e., communities that are hit first and worst by harms of environmental damage) reject offsetting as a real climate solution. Offsetting is often considered a “greenwashing” solution because it ignores community effects of fossil fuel extraction such as public health impacts from air, water, and soil pollution, biodiversity loss, and loss of land sovereignty.^53^^–^^56^ Instead, academic medical centers should focus on implementing solutions to reduce actual emissions and collaborate with host institutions to identify, develop, and fund climate solutions in the host country, as noted in the ASTMH Green Task Force recommendations.

## ENACTING SYSTEMS CHANGE GLOBALLY

In addition to reducing emissions, academic medical centers and their associated educators, clinicians, and researchers should engage in broader climate advocacy efforts. STEGH agreements could incorporate climate and health-associated infrastructure development as identified by the host institution as a component of the partnership. For example, funding a transition to renewable energy such as solar panels might provide reliable electricity and uninterrupted patient care while also improving air quality, mitigating climate change, and providing health co-benefits. Academic institutions can also deepen their interdepartmental collaboration to support novel host country initiatives. United efforts by environmental science, engineering, and agriculture departments to support host site programs addressing energy and food systems would have a greater impact than single siloed projects. In addition, through bidirectional learning, academic medical centers could develop and implement sustainability practices that fit the local context of host countries.

Taking influence to the broadest scale, healthcare workers should be empowered to advocate for action to mitigate climate change and develop climate-resilient communities.^55^ Global health professionals, in particular, have a unique opportunity to elevate the voices of those most impacted in low- and middle-income countries and actively advocate for public policy change targeting actual emissions reductions. Academic programs can create opportunities for learners and health professionals to engage in climate advocacy on local and national stages, such as organizing legislative “lobby days” and participation in the annual United Nations Conference of Parties conferences.

## CONCLUSION

Academic medical centers in HICs that sponsor STEGH should recognize the intersectionality of colonialism and medical voluntourism with the inequitable burdens of climate damage and ongoing contributions to carbon emissions. STEGH are a valuable tool for medical education; however, academic medical centers should recognize the ethical conflict of duty that international travel for global health training poses. Principles of “decolonization” and “decarbonization” should guide academic medical centers collaborating with host institutions to reconceptualize STEGH in three major ways. First, programs should restructure rotations to promote equitable partnerships that foster education and minimize carbon emissions, such as through longer rotation duration, virtual interactions, and halting international travel that is of little value to host institutions. Second, programs should include carbon literacy and acknowledge the ecological impact of STEGH, such as through an internal carbon emissions analysis that can frame impact and accountability for programs. Finally, academic medical centers should leverage their considerable resources and personnel to advocate on the global stage and invest in proposed solutions from host institutions that can address climate-induced impacts on health.
